# Supplementary figures and images for: Meiotic cells escape prolonged spindle checkpoint activity through kinetochore silencing and slippage
Source: PLoS Genet. 2023 Apr 5;19(4):e1010707. doi: 10.1371/journal.pgen.1010707 (PMC10109492; doi:10.1371/journal.pgen.1010707)

Figure S1

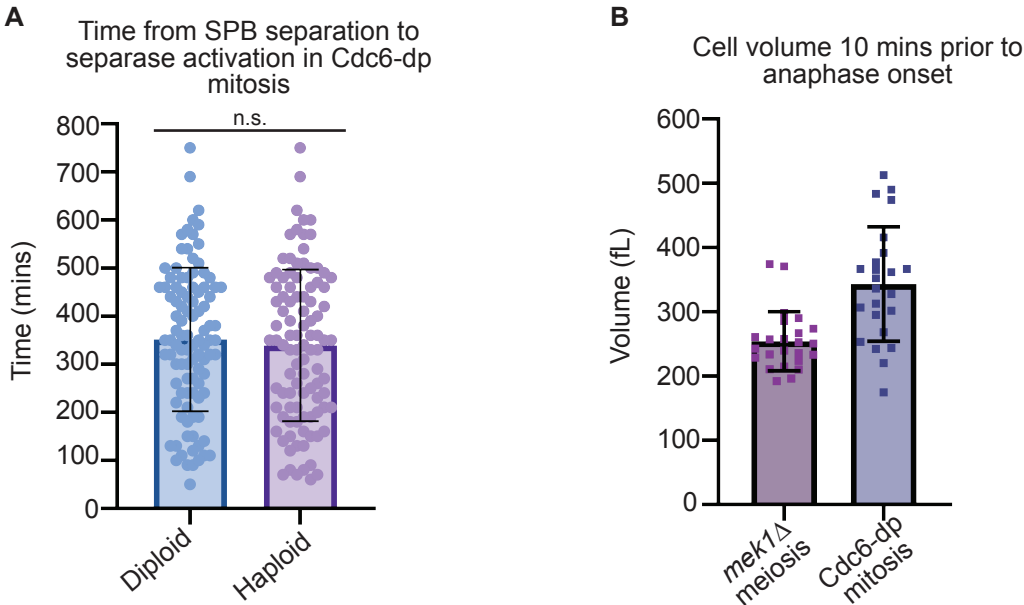

Supplement: S1 Fig — A) Graph of the time from SPB separation to dispersal of separase biosensor focus in diploid and haploid cells that are depleted of Cdc6. n≥ 100 cells per strain. n.s., not significant (Mann-Whitney test; p> 0.05). B) Graph showing cell volume measured 10 minutes prior to anaphase onset in mek1Δ meiosis and Cdc6-dp mitosis. Error bars indicate SD. n = 25 cells per genotype. (PDF) [file pgen.1010707.s001.pdf]

Figure S2

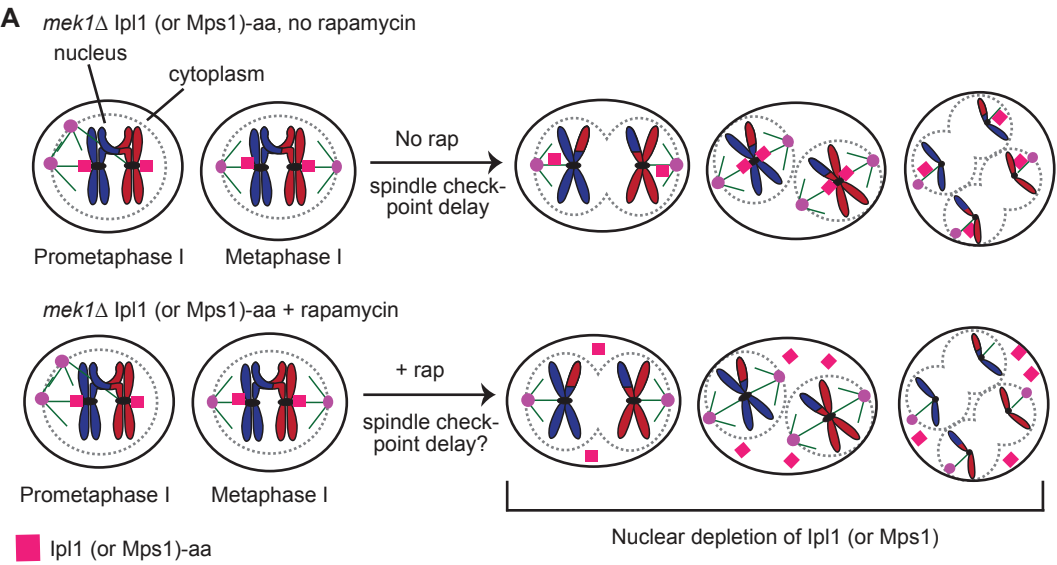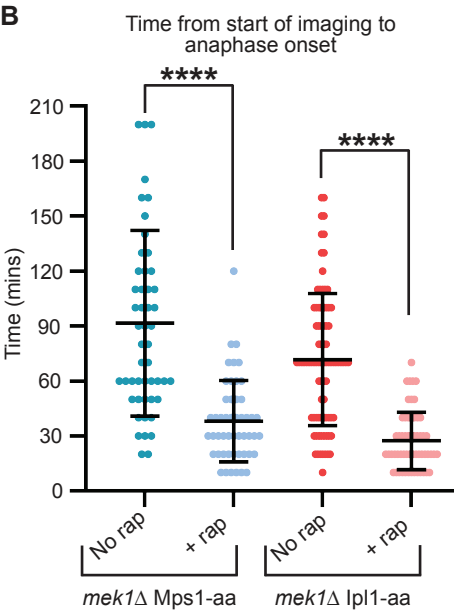

Supplement: S2 Fig — A) Cartoon of experimental design. Ipl1-aa mek1Δ and Mps1-aa mek1Δ strains progressed into metaphase I, and were then treated with rapamycin to deplete Ipl1 or Mps1 from the nucleus. Live-cell imaging began immediately following rapamycin treatment, and time from start of imaging to anaphase spindle elongation was measured. B) Graph showing time from start of imaging to anaphase onset in individual cells. Center bar is mean, and error bars represent SD. Rap = Rapamycin; aa = anchor away. n≥50 cells per genotype. Asterisk shows statistical significance between the indicated conditions (Welch’s t-test, p < 0.0001). (PDF) [file pgen.1010707.s002.pdf]

Figure S3

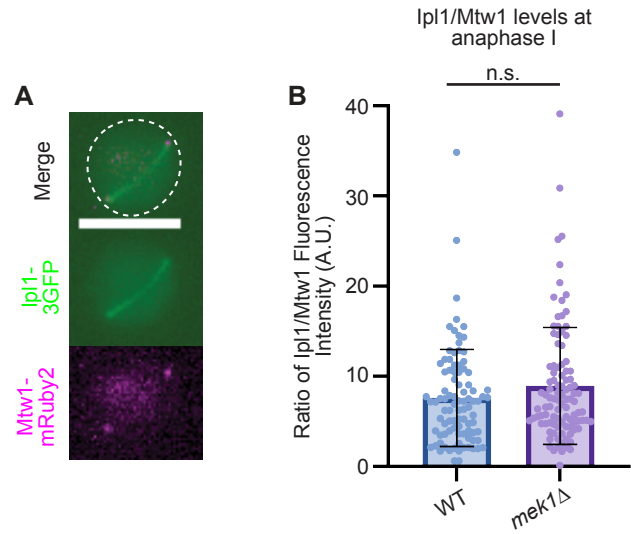

Supplement: S3 Fig — A) Representative images of a mek1Δ cell at anaphase I onset. Measurements were made by drawing circles around the Mtw1-mRuby2 focus (representing the kinetochore) and recording both Mtw1-mRuby2 and Ipl1-3GFP fluorescence intensity. Scale bar = 5 μm. B) Quantification of Ipl1/Mtw1 fluorescence intensity. 90 or more cells per genotype were scored, and the mean is plotted. Mann-Whitney test was performed and showed no statistically significant difference between WT and mek1Δ. Error bars show SD. (PDF) [file pgen.1010707.s003.pdf]

Figure S4

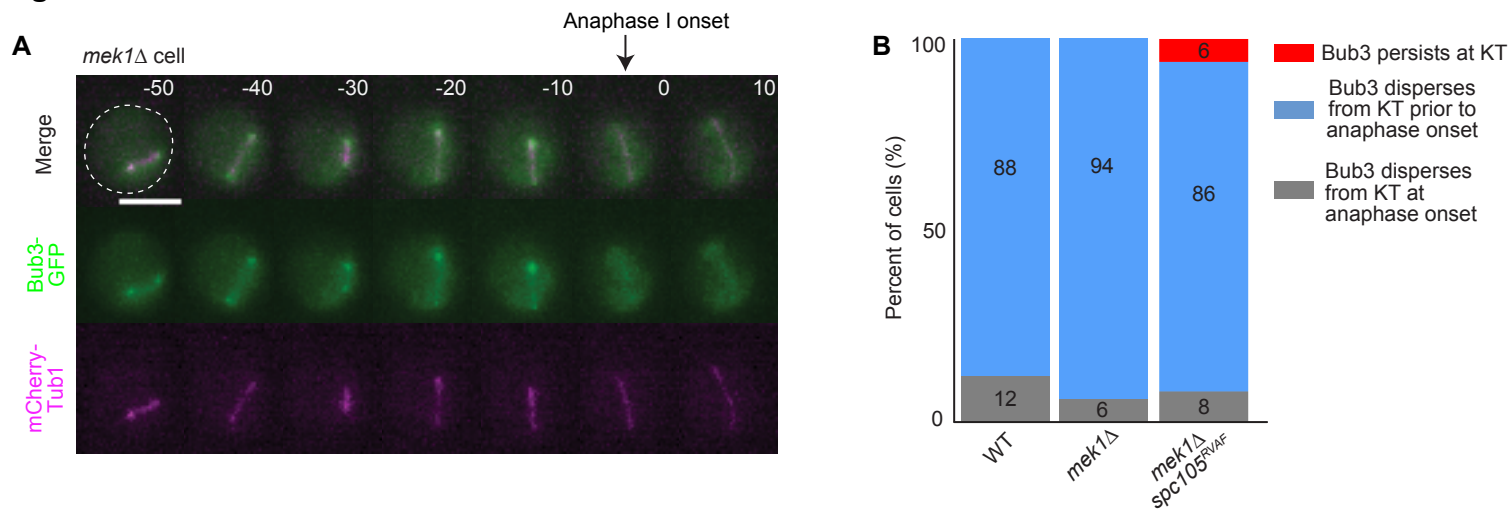

Supplement: S4 Fig — A) Time-lapse images of a mek1Δ cell show Bub3-eGFP and mCherry-Tub1 during meiosis I. Time 0 is set as the time frame in which anaphase onset occurs. Scale bar = 5 μm. B) Quantification of Bub3-eGFP dispersal in WT and mek1Δ cells. n≥0 cells per genotype. (PDF) [file pgen.1010707.s004.pdf]

Figure S5

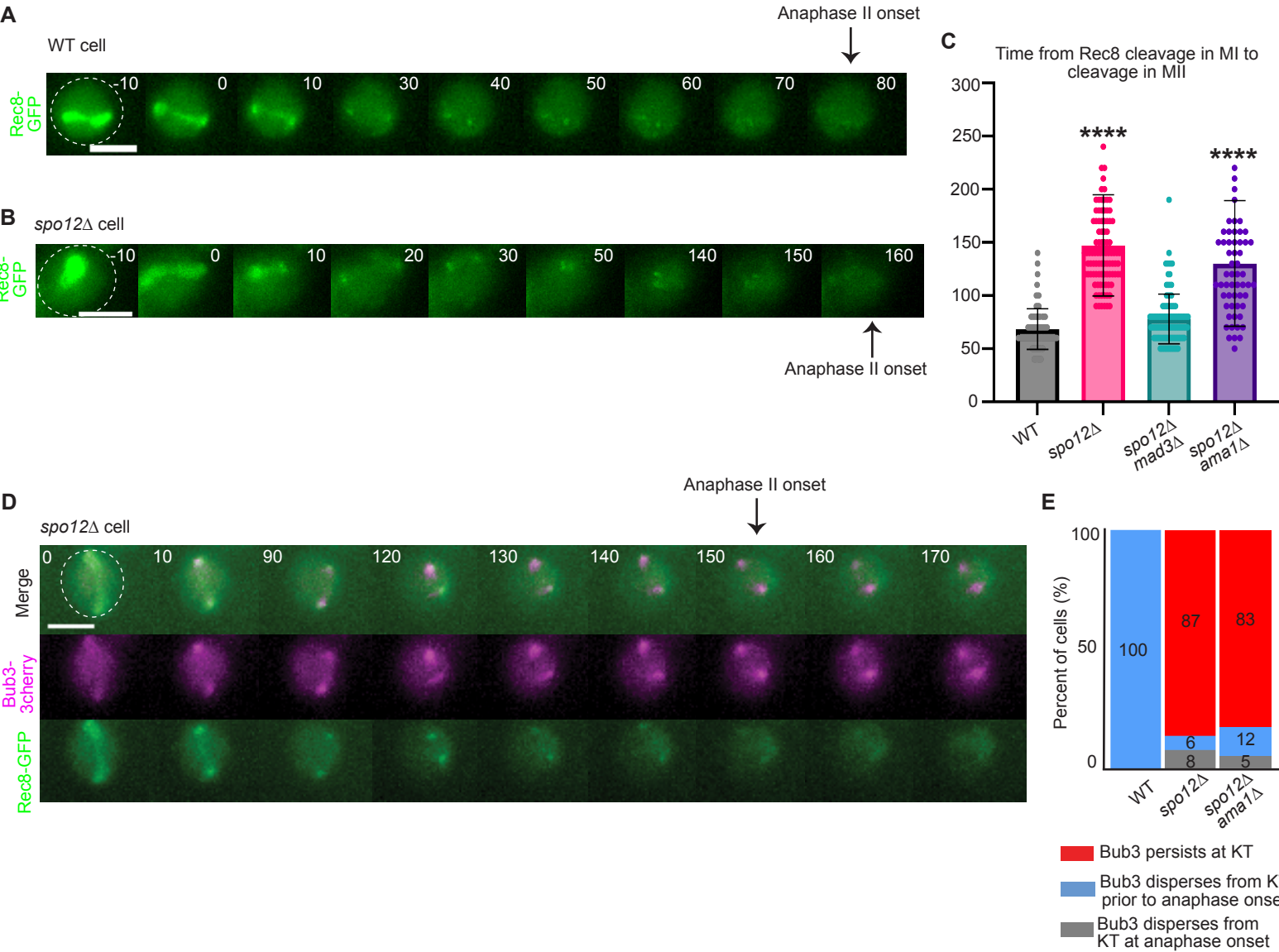

Supplement: S5 Fig — A-B) Representative time-lapse images of WT and spo12Δ cells expressing Rec8-GFP. Time 0 is the time at which Rec8 cleavage initiates in meiosis I. Scale bar = 5 μm. Error bars show SD. C) Quantification of the duration from Rec8 cleavage in meiosis I to Rec8 cleavage in meiosis II. Statistical significance was determined by Mann-Whitney test (p < 0.0001). Asterisk indicates statistically significant difference between wildtype and the indicated genotypes. n≥ 50 cells per genotype. D) Time-lapse images of cells with Rec8-GFP and Bub3-3mcherry. Time 0 is the time at which Rec8 is cleaved in meiosis I. E) Quantification of Bub3-3mCherry dispersal. n≥50 cells per genotype. (PDF) [file pgen.1010707.s005.pdf]
