# Supplementary material for: Meiotic cells escape prolonged spindle checkpoint activity through kinetochore silencing and slippage
Source: PLoS Genet. 2023 Apr 5;19(4):e1010707. doi: 10.1371/journal.pgen.1010707 (PMC10109492; doi:10.1371/journal.pgen.1010707)
Supplement: S1 Table — (PDF) [file pgen.1010707.s006.pdf]

Table S1. Budding yeast strains used in this study

Strain number:	Strain genotype:
LY3022	MATa/α Spc42-mCherry:kanMX6/+, P_{TUB1}GFP-TUB1:LEU2/ P_{TUB1}GFP-TUB1:LEU2, ZIP1-GFP/+
LY5451	MAT a, Spc42-mCherry:HphMX, LacO:TRP1, P_{CUP1}- GFP-Scc1-LacI:HIS3
LY5512	MAT a, Spc42-mCherry:HphMX, LacO:TRP1, P_{CUP1}- GFP-Scc1-LacI:HIS3, mad3::KanMX
LY8248	MAT a/α, Spc42-mCherry:HphMX /+, P_{CUP1}- GFP-Rec8-LacI:HIS3/+, LacO:TRP1 at 800 kb from CEN4/ LacO:TRP1 at 800 kb from CEN4, P_{GAL1-1}-Ndt80:TRP1/ P_{GAL1-1}-Ndt80:TRP1, Gal4-ER:URA3/ Gal4-ER:URA3
LY8312	MAT a/α, Spc42-mCherry:HphMX /+, P_{CUP1}- GFP-Rec8-LacI:HIS3/+, LacO:TRP1 at 800 kb from CEN4/ LacO:TRP1 at 800 kb from CEN4, P_{GAL1-1}-Ndt80:TRP1/ P_{GAL1-1}-Ndt80:TRP1, Gal4-ER:URA3/Gal4-ER:URA3, mad3::KanMX/mad3::KanMX
LY5657	MAT a/α, Spc42-mCherry:HphMX /+, LacO:TRP1/LacO:TRP1, P_{CUP1}-GFP-Scc1-LacI:HIS3/ P_{CUP1}-GFP-Scc1-LacI:HIS3
LY10083	MAT a/α, Spc42-mCherry:HphMX /+, LacO:TRP1/LacO:TRP1, P_{CUP1}-GFP-Scc1-LacI:HIS3/ P_{CUP1}-GFP-Scc1-LacI:HIS3, mad3::KanMX/mad3::KanMX
LY5661	MAT a/α, Spc42-mCherry:HphMX /+, cdc6::HISG/cdc6::HISG, P_{GAL1-1}-UbiCdc6:URA3/ P_{GAL1-1}-UbiCdc6:URA3, PDS1:Pds1myc18:Leu2/pds1:Pds1myc18:Leu2, LacO:TRP1/ LacO:TRP1, P_{CUP1}-GFP-Scc1-LacI:HIS3/ P_{CUP1}-GFP-Scc1-LacI:HIS3
LY10033	MAT a/α, Spc42-mCherry:HphMX /+, cdc6::HISG/cdc6::HISG, P_{GAL1-1}-UbiCdc6:URA3/ P_{GAL1-1}-UbiCdc6:URA3, PDS1:Pds1myc18:Leu2/pds1:Pds1myc18:Leu2, LacO:TRP1/ LacO:TRP1, P_{CUP1}-GFP-Scc1-LacI:HIS3/ P_{CUP1}-GFP-Scc1-LacI:HIS3, mad3::KanMX/mad3::KanMX
LY5658	MAT a/α, Spc42-mCherry:HphMX /+, P_{CUP1}- GFP-Rec8-LacI:HIS3/ P_{CUP1}- GFP-Rec8-LacI:HIS3, LacO:TRP1 at 800 kb from CEN4/ LacO:TRP1 at 800 kb from CEN4
LY8211	MAT a/α, Spc42-mCherry:HphMX /+, P_{CUP1}- GFP-Rec8-LacI:HIS3/ P_{CUP1}- GFP-Rec8-LacI:HIS3, LacO:TRP1 at 800 kb from CEN4/ LacO:TRP1 at 800 kb from CEN4, mad3::KanMX/mad3::KanMX

LY5659	MAT a/α, Spc42-mCherry:HphMX /+, P_{CUP1}- GFP-Rec8-LacI:HIS3/ P_{CUP1}- GFP-Rec8-LacI:HIS3, LacO:TRP1 at 800 kb from CEN4/ LacO:TRP1 at 800 kb from CEN4, spo11::NatMX/spo11::NatMX, spo11-Y135F:URA3/ spo11-Y135F:URA3
LY5660	MATa/α, Spc42-mCherry:HphMX /+, P_{CUP1}- GFP-Rec8-LacI:HIS3/ P_{CUP1}- GFP-Rec8-LacI:HIS3, LacO:TRP1 at 800 kb from CEN4/ LacO:TRP1 at 800 kb from CEN4, spo11::NatMX/spo11::NatMX, spo11-Y135F:URA3/ spo11-Y135F:URA3, mad3::KanMX/mad3::KanMX
LY10068	MAT a/α, Spc42-mCherry:HphMX /+, P_{CUP1}- GFP-Rec8-LacI:HIS3/ P_{CUP1}- GFP-Rec8-LacI:HIS3, LacO:TRP1 at 800 kb from CEN4/ LacO:TRP1 at 800 kb from CEN4, mek1::KanMX/mek1::KanMX
LY10082	MATa/α, Spc42-mCherry:HphMX /+, P_{CUP1}- GFP-Rec8-LacI:HIS3/ P_{CUP1}- GFP-Rec8-LacI:HIS3, LacO:TRP1 at 800 kb from CEN4/ LacO:TRP1 at 800 kb from CEN4, mek1::NatMX/mek1::NatMX, mad3::KanMX/mad3::KanMX
LY8140	MATa/α, Spc42-mCherry:HphMX /+, P_{CUP1}- GFP-Rec8-LacI:HIS3/ P_{CUP1}- GFP-Rec8-LacI:HIS3, LacO:TRP1 at 800 kb from CEN4/ LacO:TRP1 at 800 kb from CEN4, Chrom V carlsbergensis[ivl1::pRM12[NatR:LacO]]/ Chrom V cerevisiae
LY8264	MATa/α, Spc42-mCherry:HphMX /+, P_{CUP1}- GFP-Rec8-LacI:HIS3/ P_{CUP1}- GFP-Rec8-LacI:HIS3, LacO:TRP1 at 800 kb from CEN4/ LacO:TRP1 at 800 kb from CEN4, Chrom V carlsbergensis[ivl1::pRM12[NatR:LacO]]/ Chrom V cerevisiae, mad3::KanMX/mad3::KanMX
LY8210	MATa/α, Spc42-mCherry:HphMX /+, P_{CUP1}- GFP-Rec8-LacI:HIS3/ P_{CUP1}- GFP-Rec8-LacI:HIS3, LacO:TRP1 at 800 kb from CEN4/ LacO:TRP1 at 800 kb from CEN4, SLC:VL107-LacO:Leu2
LY8212	MATa/α, Spc42-mCherry:HphMX /+, P_{CUP1}- GFP-Rec8-LacI:HIS3/ P_{CUP1}- GFP-Rec8-LacI:HIS3, LacO:TRP1 at 800 kb from CEN4/ LacO:TRP1 at 800 kb from CEN4, mad3::KanMX/mad3::KanMX, SLC:VL107-LacO:Leu2
LY8156	MATa/α, Spc42-mCherry:HphMX /+, P_{CUP1}- GFP-Rec8-LacI:HIS3/ P_{CUP1}- GFP-Rec8-LacI:HIS3, LacO:TRP1 at 800 kb from CEN4/ LacO:TRP1 at 800 kb from CEN4, Chrom V carlsbergensis[ivl1::pRM12[NatR:LacO]]/ Chrom V cerevisiae, SLC:VL107-LacO:Leu2
LY8303	MATa/α, Spc42-mCherry:HphMX /+, P_{CUP1}- GFP-Rec8-LacI:HIS3/ P_{CUP1}- GFP-Rec8-LacI:HIS3, LacO:TRP1 at 800 kb from CEN4/ LacO:TRP1 at 800 kb from CEN4, Chrom V carlsbergensis[ivl1::pRM12[NatR:LacO]]/ Chrom V cerevisiae, mad3::KanMX, mad3::KanMX, SLC:VL107-LacO:Leu2

LY4415	MATa/α, Spc42-mCherry: HphMX/+, P_{CUP1}-LACI-GFP:HIS3/ P_{CYC1}-LACI-GFP:HIS3, LacO:TRP/ LacO:TRP
LY4568	MATa/α, Spc42-mCherry: HphMX/+, P_{CUP1}-LACI-GFP:HIS3/ P_{CUP1}-LACI-GFP:HIS3, LacO:TRP/ LacO:TRP, mad3::KanMX/mad3::KanMX
LY4515	MATa/α, Spc42-mCherry: HphMX/+, P_{CUP1}-LACI-GFP:HIS3/ P_{CUP1}-LACI-GFP:HIS3, LacO:TRP/ LacO:TRP, mek1::KanMX/mek1::KanMX
LY4607	MATa/α, Spc42-mCherry: HphMX/+, P_{CUP1}-LACI-GFP:HIS3/ P_{CUP1}-LACI-GFP:HIS3, LacO:TRP/ LacO:TRP, mek1::KanMX/mek1::KanMX, mad3::KanMX/mad3::KanMX
LY10086	MATa/α, P_{CUP1}- GFP-Rec8-LacI:HIS3/ P_{CUP1}- GFP-Rec8-LacI:HIS3, LacO:TRP1 at 800 kb from CEN4/ LacO:TRP1 at 800 kb from CEN4, bub3::Leu2/bub3::Leu2, trp1::Bub3-3mCherry:TRP1/ trp1::Bub3-3mCherry:TRP1
LY10067	MATa/α, P_{CUP1}- GFP-Rec8-LacI:HIS3/ P_{CUP1}- GFP-Rec8-LacI:HIS3, LacO:TRP1 at 800 kb from CEN4/ LacO:TRP1 at 800 kb from CEN4, bub3::Leu2/bub3::Leu2, trp1::Bub3-3mCherry:TRP1/ trp1::Bub3-3mCherry:TRP1, mek1::KanMX/mek1::KanMX
LY10066	MATa/α, P_{CUP1}- GFP-Scc1-LacI:HIS3/ P_{CUP1}- GFP-Scc1-LacI:HIS3, LacO:TRP1 at 800 kb from CEN4/ LacO:TRP1 at 800 kb from CEN4, bub3::Leu2/bub3::Leu2, trp1::Bub3-3mCherry:TRP1/ trp1::Bub3-3mCherry:TRP1
LY10036	MATa/α, P_{CUP1}- GFP-Scc1-LacI:HIS3/ P_{CUP1}- GFP-Scc1-LacI:HIS3, LacO:TRP1 at 800 kb from CEN4/ LacO:TRP1 at 800 kb from CEN4, bub3::Leu2/bub3::Leu2, trp1::Bub3-3mCherry:TRP1/ trp1::Bub3-3mCherry:TRP1, cdc6::HISG/cdc6::HISG, P_{GAL1-1}-UbiCdc6:URA3/ P_{GAL1-1}-UbiCdc6:URA3, PDS1:Pds1myc18:Leu2/pds1:Pds1myc18:Leu2
LY9241	MATa/α, P_{CUP1}- GFP-Rec8-LacI:HIS3/ P_{CUP1}- GFP-Rec8-LacI:HIS3, LacO:TRP1 at 800 kb from CEN4/ LacO:TRP1 at 800 kb from CEN4, bub3::Leu2/bub3::Leu2, trp1::Bub3-3mCherry:TRP1/ trp1::Bub3-3mCherry:TRP1, P_{Gal1}-Ndt80:TRP1/ P_{Gal1}-Ndt80:TRP1, Gal4-ER:URA3/ Gal4-ER:URA3
LY9417	MATa/α, tor1-1:HIS3/tor1-1:HIS3, fpr1::natMX4/fpr1::natMX4, RPL13A-2XFKBP12:TRP1/RPL13A-2XFKBP12:TRP1, Spc42-mCherry:HphMX/+, P_{TUB1}GFP-TUB1:URA3/P_{TUB1}GFP-TUB1:URA3, Spc105-FRB:KanMX/Spc105-FRB:KanMX, P_{Spc105}-Spc105:LEU2/P_{Spc105}-Spc105:LEU2
LY10085	MATa/α, tor1-1:HIS3/tor1-1:HIS3, fpr1::natMX4/fpr1::natMX4, RPL13A-2XFKBP12:TRP1/RPL13A-2XFKBP12:TRP1, Spc42-mCherry:HphMX/+,

	P_{TUB1}GFP-TUB1:URA3/P_{TUB1}GFP-TUB1:URA3, Spc105-FRB:KanMX/Spc105-FRB:KanMX, P_{Spc105}-spc105^{RASA}:LEU2/P_{Spc105}-spc105^{RASA}:LEU2
LY10087	MATa/α, tor1-1:HIS3/tor1-1:HIS3, fpr1::natMX4/fpr1::natMX4, RPL13A-2XFKBP12:TRP1/RPL13A-2XFKBP12:TRP1, Spc42-mCherry:HphMX/+, P_{TUB1}GFP-TUB1:URA3/P_{TUB1}GFP-TUB1:URA3, Spc105-FRB:KanMX/Spc105-FRB:KanMX, P_{Spc105}-spc105^{RASA}:LEU2/P_{Spc105}-spc105^{RASA}:LEU2, mad3::KanMX/mad3::KanMX
LY9533	MATa/α, tor1-1:HIS3/tor1-1:HIS3, fpr1::natMX4/fpr1::natMX4, RPL13A-2XFKBP12::loxP /RPL13A-2XFKBP12::loxP, Spc42-mCherry:HphMX/+, Bub3-eGFP:TRP1/ Bub3-eGFP:TRP1, Spc105-FRB:KanMX/Spc105-FRB:KanMX, P_{Spc105}-Spc105:LEU2/P_{Spc105}-Spc105:LEU2
LY10084	MATa/α, tor1-1:HIS3/tor1-1:HIS3, fpr1::natMX4/fpr1::natMX4, RPL13A-2XFKBP12::loxP /RPL13A-2XFKBP12::loxP, Spc42-mCherry:HphMX/+, Bub3-eGFP:TRP1/ Bub3-eGFP:TRP1, Spc105-FRB:KanMX/Spc105-FRB:KanMX, P_{Spc105}-Spc105^{RASA}:LEU2/P_{Spc105}-Spc105^{RASA}:LEU2
LY3022	MATa/α Spc42-mCherry:kanMX6/+, P_{TUB1}GFP-TUB1:LEU2/ P_{TUB1}GFP-TUB1:LEU2, ZIP1-GFP/+
LY5855	MATa/α, tor1-1::HIS3/tor1-1, fpr1::NatMX/fpr1::natMX4, RPL13A-2XFKBP12:TRP1/RPL13A-2XFKBP12:TRP1, Spc42-mCherry:HphMX/+, P_{TUB1}GFP-TUB1:URA3/P_{TUB1}GFP-TUB1:URA3, Spc105-FRB:KanMX/Spc105-FRB:KanMX, P_{Rec8}-Spc105:LEU2/P_{Rec8}-Spc105:LEU2
LY10110	MATa/α, tor1-1:HIS3/tor1-1:HIS3, fpr1::NatMX/fpr1::natMX4, RPL13A-2XFKBP12:TRP1/RPL13A-2XFKBP12:TRP1, Spc42-mCherry:HphMX/+, P_{TUB1}GFP-TUB1:URA3/P_{TUB1}GFP-TUB1:URA3, Spc105-FRB:KanMX/Spc105-FRB:KanMX, P_{Rec8}-Spc105^{RASA}:LEU2/P_{Rec8}-Spc105^{RASA}:LEU2, mek1::KanMX/mek1::KanMX
LY6995	MATa/α, tor1-1:HIS3/tor1-1:HIS3, fpr1::NatMX/fpr1::natMX4, RPL13A-2XFKBP12:TRP1/RPL13A-2XFKBP12:TRP1, Spc42-mCherry:HphMX/+, P_{TUB1}GFP-TUB1:URA3/P_{TUB1}GFP-TUB1:URA3, Spc105-FRB:KanMX/Spc105-FRB:KanMX, P_{Rec8}-Spc105^{RASA}:LEU2/P_{Rec8}-Spc105^{RASA}:LEU2, mad3::KanMX/mad3::KanMX
LY2606	MATa/α mek1::KanMX6/ mek1::KanMX6, Spc42-mCherry:HphMX/+, P_{TUB1}GFP-TUB1:LEU2/ P_{TUB1}GFP-TUB1:LEU2, ZIP1-GFP/+
LY10111	MATa/α, tor1-1:HIS3/tor1-1:HIS3, fpr1::NatMX/fpr1::natMX4, RPL13A-2XFKBP12:TRP1/RPL13A-2XFKBP12:TRP1, Spc105-FRB:KanMX/Spc105-

	FRB:KanMX, P_{Rec8}-Spc105^{RASA}:LEU2/P_{Rec8}-Spc105^{RASA}:LEU2, Bub3-eGFP:TRP1/Bub3-eGFP:TRP1, Spc42-mCherry:HphMX/+, mek1::HphMX/mek1::HphMX
LY9592	MATa/α, tor1-1:HIS3/tor1-1:HIS3, fpr1::NatMX/fpr1::natMX4, RPL13A-2XFKBP12::loxP/RPL13A-2XFKBP12::loxP, Spc105-FRB:KanMX/Spc105-FRB:KanMX, P_{Rec8}-Spc105^{RASA}:LEU2/P_{Rec8}-Spc105^{RASA}:LEU2, Bub3-eGFP:TRP1/Bub3-eGFP:TRP1, Spc42-mCherry:HphMX/+
LY9946	MATa/α, Spc42-mCherry:HphMX /+, P_{CUP1}- GFP-Rec8-LacI:HIS3/ P_{CUP1}- GFP-Rec8-LacI:HIS3, LacO:TRP1 at 800 kb from CEN4/ LacO:TRP1 at 800 kb from CEN4, mek1::KanMX/mek1::KanMX, *spc105*^{RVAF}*/spc105*^{RVAF}
LY10032	MATa/α, Spc42-mCherry:HphMX /+, P_{CUP1}- GFP-Scc1-LacI:HIS3/ P_{CUP1}- GFP-Scc1-LacI:HIS3, LacO:TRP1/ LacO:TRP1, Spc42-mCherry:HphMX/+, cdc6::HisG/cdc6::HisG, P_{Gal1}-ubiCdc6:URA3/ P_{Gal1}-ubiCdc6:URA3, *spc105*^{RVAF}*/spc105*^{RVAF}
LY10092	MATa/α, Spc42-mCherry:HphMX /+, P_{CUP1}- GFP-Scc1-LacI:HIS3/ P_{CUP1}- GFP-Scc1-LacI:HIS3, LacO:TRP1 at 800 kb from CEN4/ LacO:TRP1 at 800 kb from CEN4, bub3::Leu2/bub3::Leu2, trp1::Bub3-3mCherry:TRP1/trp1::Bub3-3mCherry:TRP1, cdc6::HisG/cdc6::HisG, P_{Gal1}-ubiCdc6:URA3/ P_{Gal1}-ubiCdc6:URA3, *spc105*^{RVAF}*/spc105*^{RVAF}
LY9351	MATa/α, tor1-1/tor1-1:HIS3, fpr1::NatMX/fpr1::natMX4, RPL13A-2XFKBP12:TRP1/RPL13A-2XFKBP12::loxP, Ipl1-FRB:KanMX/Ipl1-FRB:KanMX, Spc42-mCherry:HphMX/+, mek1::HphMX/mek1::HIS3MX
LY10112	MAT a/α, tor1-1/tor1-1, fpr1::NatMX/fpr1::natMX4, RPL13A-2XFKBP12::loxP/RPL13A-2XFKBP12::loxP, Mps1-FRB:KanMX/Mps1-FRB:KanMX, Spc42-mCherry:HphMX/+, mek1::HIS3MX/mek1::HIS3MX
LY10113	MAT a/α, Ipl1-3GFP:NatMX/Ipl1-3GFP:NatMX, Mtw1-mRuby2:His5/Mtw1-mRuby2:His5
LY10114	MAT a/α, Ipl1-3GFP:NatMX/Ipl1-3GFP:NatMX, Mtw1-mRuby2:His5/Mtw1-mRuby2:His5, mek1::KanMX/mek1::KanMX
LY5857	MAT a/α, Bub3-eGFP:TRP1/Bub3-eGFP:TRP1, Spc42-mCherry:hph/+
LY9670	MAT a/α, Bub3-eGFP:TRP1/Bub3-eGFP:TRP1, mCherry-Tub1:ADE2/mCherry-Tub1:ADE2, mCherry-Tub1:URA3/mCherry-Tub1:URA3

LY9669	MAT a/α, Bub3-eGFP:TRP1/Bub3-eGFP:TRP1, mCherry-Tub1:ADE2/mCherry-Tub1:ADE2, mCherry-Tub1:URA3/mCherry-Tub1:URA3, mek1::NatMX/mek1::NatMX
LY5791	MATa/α, Spc42-mCherry:HphMX/+, Mad2-3GFP:KanMX/Mad2-3GFP:KanMX
LY8397	MATa/α, Spc42-mCherry:HphMX/+ Rec8-yEGFP:KanMX/ Rec8-yEGFP:KanMX, spo12::HIS3MX/spo12::HIS3MX
LY8473	MATa/α, Spc42-mCherry:HphMX/+, Rec8-yEGFP:KanMX/ Rec8-yEGFP:KanMX, spo12::HIS3MX/spo12::HIS3MX, mad3::KanMX/mad3::KanMX
LY10046	MATa/α, Spc42-mCherry:HphMX/+, Rec8-yEGFP:KanMX/ Rec8-yEGFP:KanMX, spo12::HIS3MX/spo12::HIS3MX, ama1::NatMX/ama1::NatMX
LY9322	MATa/α, Spc42-mCherry:HphMX/+, Rec8-yEGFP:KanMX/ Rec8-yEGFP:KanMX
LY8599	MATa/α, Spc42-mCherry:HphMX/+, Rec8-yEGFP:KanMX/ Rec8-yEGFP:KanMX, bub3::Leu2/bub3::Leu2, trp1::Bub3-3mCherry:TRP1/ trp1::Bub3-3mCherry:TRP1
LY8598	MATa/α, Spc42-mCherry:HphMX/+, Rec8-yEGFP:KanMX/ Rec8-yEGFP:KanMX, bub3::Leu2/bub3::Leu2, trp1::Bub3-3mCherry:TRP1/ trp1::Bub3-3mCherry:TRP1, spo12::HIS3MX/spo12::HIS3MX
LY10047	MATa/α, Spc42-mCherry:HphMX/+, Rec8-yEGFP:KanMX/ Rec8-yEGFP:KanMX, bub3::Leu2/bub3::Leu2, trp1::Bub3-3mCherry:TRP1/ trp1::Bub3-3mCherry:TRP1, spo12::HIS3MX/spo12::HIS3MX, ama1::HphMX/ama1::HphMX
