# Supplementary material for: Meiotic cells escape prolonged spindle checkpoint activity through kinetochore silencing and slippage
Source: PLoS Genet. 2023 Apr 5;19(4):e1010707. doi: 10.1371/journal.pgen.1010707 (PMC10109492; doi:10.1371/journal.pgen.1010707)
Supplement: S2 Table — (PDF) [file pgen.1010707.s007.pdf]

Table S2. Plasmids used in this study

Plasmid name	Relevant subcloned regions	Purpose	Source
pLB304	P_{Cup1}-GFP-Scc1-LacI-mCherry:His	Mitosis separase biosensor	[1]
pLB305	P_{Cup1}-GFP-Rec8-LacI:His	Meiosis separase biosensor	[1]
pSLB22	LacO:Trp1	LacO repeats at the *TRP1* locus	[2]
pLB113	P_{CUP1}-LacI-GFP:His3	LacI-GFP under the *CUP1* promoter at the *HIS3* locus	[2]
pLB358	P_{SPC105}-SPC105$^{WT:}$Leu2	Spc105 under its endogenous promoter at the *LEU2* locus	This study
pLB300	P_{Rec8}-SPC105^{WT} at Trp1	Spc105 under the Rec8 promoter at the *TRP1* locus	This study
pLB321	P_{Rec8}-SPC105^{WT} at Leu2	Spc105 under the Rec8 promoter at the *LEU2* locus	This study
pLB365	P_{SPC105}-SPC105^{RASA} at Leu2	*spc105^{RASA}* under its endogenous promoter at the *LEU2* locus	This study
pLB302	P_{Rec8}-SPC105^{RASA} at Trp1	*spc105^{RASA}* under the Rec8 promoter at the *TRP1* locus	This study
pLB318	P_{Rec8}-SPC105^{RASA} at Leu2	*spc105^{RASA}* under the Rec8 promoter at the *LEU2* locus	This study

References:

1. Yaakov G, Thorn K, Morgan DO. Separase biosensor reveals that cohesin cleavage timing depends on phosphatase PP2A(Cdc55) regulation. Developmental Cell. 2012;23(1):124-36. Epub 2012/07/21. doi: 10.1016/j.devcel.2012.06.007. PubMed PMID: 22814605; PubMed Central PMCID: PMCPMC3413326.
2. Straight AF, Belmont AS, Robinett CC, Murray AW. GFP tagging of budding yeast chromosomes reveals that protein-protein interactions can mediate sister chromatid cohesion. Current Biology : CB. 1996;6(12):1599-608. Epub 1996/12/01. doi: 10.1016/s0960-9822(02)70783-5. PubMed PMID: 8994824.
