# Supplementary material for: Meiotic cells escape prolonged spindle checkpoint activity through kinetochore silencing and slippage
Source: PLoS Genet. 2023 Apr 5;19(4):e1010707. doi: 10.1371/journal.pgen.1010707 (PMC10109492; doi:10.1371/journal.pgen.1010707)
Supplement: S3 Table — (PDF) [file pgen.1010707.s008.pdf]

Table S3. List of reagents used in this study

Reagent	Source	Identifier
Yeast extract	Thermo Fisher Scientific	Ref#212720, Lot#2352585
Peptone	Thermo Fisher Scientific	Ref#211820, Lot#1263496
Dextrose (D-Glucose) Anhydrous	Fisher Chemical	Lot#204841
Potassium acetate	Fisher Bioreagents	Lot#210889
Yeast nitrogen base without amino acids	Thermo Fisher Scientific	Ref#291920, Lot#9148845
Synthetic complete mixture drop-out: Complete	Formedium	Ref# DSCK2500, Batch# FM0A416/006650
Bacto-agar	Thermo Fisher Scientific	Cas#: 214010
β-estradiol	Sigma	Cas#: 50-28-2
Rapamycin	Fisher BioReagents	Cas#: 53123-88-9
Alpha factor	Zymo research	Cas#: Y1001

Nocodazole	Sigma	Cat# M1404
Concanavalin A	Sigma	Cas#: 11028-71-0
NaCl	Sigma	Cat# S5886-500G
Gentamicin	Sigma	Cat# G1272-10ML
Nourseothricin Sulfate (Clonat)	Thermo Fisher	Cat# 50-103-5835
Hygromycin B	Cornig	Product # 30-240-CR
Dimethyl sulfoxide (DMSO)	Sigma	Cat#472301
Ethanol	Fisher	Cas#64-17-5
